# Knee Joint Distraction Compared to Total Knee Arthroplasty for Treatment of End Stage Osteoarthritis: Simulating Long-Term Outcomes and Cost-Effectiveness

**DOI:** 10.1371/journal.pone.0155524

**Published:** 2016-05-12

**Authors:** J. A. D. van der Woude, S. C. Nair, R. J. H. Custers, J. M. van Laar, N. O. Kuchuck, F. P. J. G. Lafeber, P. M. J. Welsing

**Affiliations:** 1 Department of Orthopedics, Maartenskliniek Woerden, Woerden, The Netherlands; 2 Department of Rheumatology & Clinical Immunology, UMC Utrecht, Utrecht, The Netherlands; 3 Department of Orthopedics, UMC Utrecht, Utrecht, The Netherlands; Mayo Clinic Minnesota, UNITED STATES

## Abstract

**Objective:**

In end-stage knee osteoarthritis the treatment of choice is total knee arthroplasty (TKA). An alternative treatment is knee joint distraction (KJD), suggested to postpone TKA. Several studies reported significant and prolonged clinical improvement of KJD. To make an appropriate decision regarding the position of this treatment, a cost-effectiveness and cost-utility analysis from healthcare perspective for different age and gender categories was performed.

**Methods:**

A treatment strategy starting with TKA and a strategy starting with KJD for patients of different age and gender was simulated. To extrapolate outcomes to long-term health and economic outcomes a Markov (Health state) model was used. The number of surgeries, QALYs, and treatment costs per strategy were calculated. Costs-effectiveness is expressed using the cost-effectiveness plane and cost-effectiveness acceptability curves.

**Results:**

Starting with KJD the number of knee replacing procedures could be reduced, most clearly in the younger age categories; especially revision surgery. This resulted in the KJD strategy being dominant (more effective with cost-savings) in about 80% of simulations (with only inferiority in about 1%) in these age categories when compared to TKA. At a willingness to pay of 20.000 Euro per QALY gained, the probability of starting with KJD to be cost-effective compared to starting with a TKA was already found to be over 75% for all age categories and over 90–95% for the younger age categories.

**Conclusion:**

A treatment strategy starting with knee joint distraction for knee osteoarthritis has a large potential for being a cost-effective intervention, especially for the relatively young patient.

## Introduction

In the event of failure of conservative treatment in generalized knee osteoarthritis the treatment of choice is often a total knee arthroplasty (TKA). TKA is now generally regarded as the gold standard for generalized knee osteoarthritis, being a safe and (cost)-effective procedure [1]. However, in younger and middle aged patients there are some legitimate concerns regarding the effectiveness of TKA related to the time to failure of TKA and need for revision surgery [2]. Younger patients (<55) have an almost five times higher risk of revision, one of main reasons being aseptic loosening [3–5]. The increasing rate of primary and revision TKAs is a considerable healthcare burden [6]. For young and middle aged patients with generalized knee osteoarthritis alternative treatment strategies are therefore needed. One of those alternatives is knee joint distraction (KJD). KJD is a surgical procedure in which an external fixation frame is used to extend the tibio-femoral joint for 6–8 weeks.

In short the distraction treatment comes down to the following; two dynamic monotubes are placed on either side of the knee joint (lateral and medial) and are fixed to femur and tibia with two bone pins each. The knee joint is distracted for ~5 mm and patients are allowed to fully load the distracted knee if needed supported with crutches. After 6–8 weeks the frame and pins are removed [7]. The scientific rationale is that full mechanical unloading of the knee joint prevents further wear and tear and enables intrinsic cartilaginous tissue repair [8]. In the past one prospective uncontrolled study was conducted, treating 20 patients originally considered for TKA. Results were promising, with prolonged clinical benefit and cartilaginous tissue repair on radiographs and magnetic resonance images [7,9]. Currently RCTs are being conducted, comparing KJD with hight tibial osteotomy and TKA [10]. To make appropriate decisions regarding the specific position of KJD for generalized knee osteoarthritis, the long-term health effects and cost-effectiveness needs to be compared to the current treatment standard for this condition (TKA). Even though currently long-term data on KJD is limited, early information on these issues can help to guide optimal implementation of KJD for patients and society, e.g. selection of patients for further studies. Therefore we set out an (early) cost-effectiveness evaluation comparing KJD with TKA from a healthcare perspective [11]. In addition, we determined the influence of age and gender in this comparison

## Materials & Methods

### Patients and treatment data

The target population for our analysis consisted of patients with advanced, generalized knee osteoarthritis indicated for TKA. However, follow-up data for KJD is still limited. Before KJD multiple studies have been conducted with joint distraction as treatment for severe ankle osteoarthritis [8]. Since ankle and knee distraction are conceptually comparable with no statistically significant difference in survival, we decided to combine these data to strengthen the modeling over a longer time, for time to KJD failure [12].

For KJD, data was derived from a feasibility study (six patients) and a prospective follow-up study (twenty patients). These 26 patients were treated with KJD between 2002 and 2008 at the University Medical Center Utrecht (UMCU). To strengthen this limited follow-up and number of patients treated, data from an open prospective multi-center study in patients who underwent distraction as a treatment for severe ankle osteoarthritis was added. Seventy-four patients underwent joint distraction of the ankle between 1993 and 2001. An overview of these studies, with mean age and survival time is given in Table 1.

**Table 1:** Overview of studies used to derive data for KJD regarding time to failure.

Type of study	Number of patients	Average age (range)	% Female	Lost to follow-up	Number of failures	Mean survival time failures (range)
Feasibility study and prospective follow-up (Knee Joint Distraction)	26	48.3 yrs (32–57 yrs)	42%	3	5	61 months (45–84 months)
Prospective multi-center study (Ankle Distraction)	74	43.3 yrs (18–65 yrs)	45%	6	25	38 months (6–120 months)

All studies were approved by the medical ethics review committee of the UMCU and all clinical investigations have been conducted according to the principles expressed in the Declaration of Helsinki. All patients gave written informed consent.

Regarding time to failure for TKA and revision TKA we used published data up to 12 years from the Australian Orthopedic Association National Joint Replacement Registry (AOANJRR) stratified by age category and gender, as no such suitable Dutch or European data (from e.g. Scandinavian registries) were available [13,14].

### Time to failure for the different treatments

**Fig 1:**
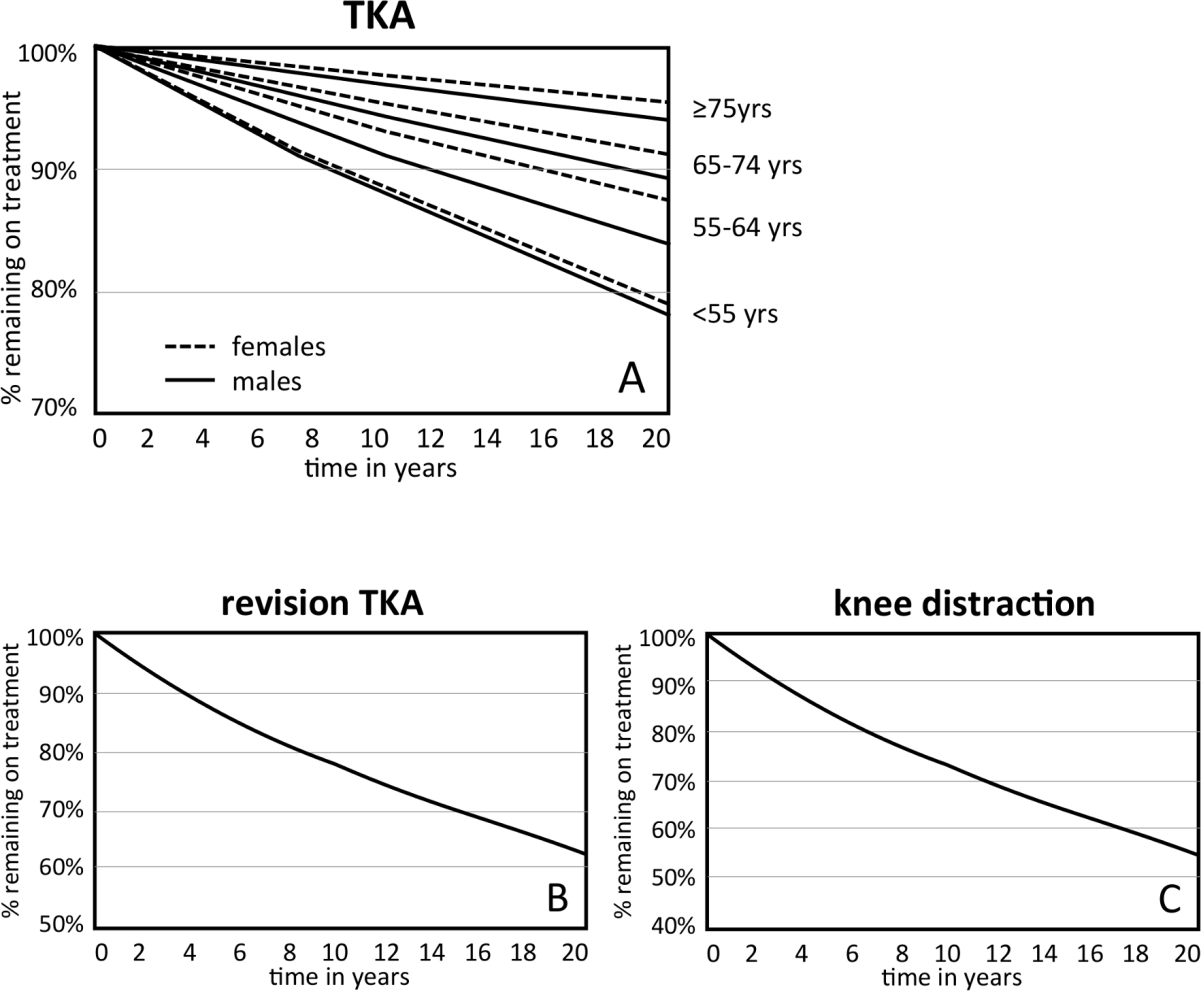
Survival curves. A Parametric Weibull distribution was fitted to extrapolate the time to failure for the different procedures (per age- gender category): for TKA **(A)**, revision TKA **(B)**, and knee distraction **(C)**.

To extrapolate the obtained short and intermediate term failure probabilities for the different treatments to long-term failure times a parametric (Weibull) regression analysis was performed on the recreated individual patient data. To fit this model as close as possible to the (published) survival curve(s), recreated individual patient data was simulated assuming no censoring [15]. Fig 1A, 1B and 1C presents survival curves based on a parametric Weibull distribution, fitted to extrapolate the time to failure for the different procedures. Time to failure was only stratified (age and gender) for TKA, as for both KJD and revision TKA no data for subgroups was available.

### Health Economic simulation model

**Fig 2:**
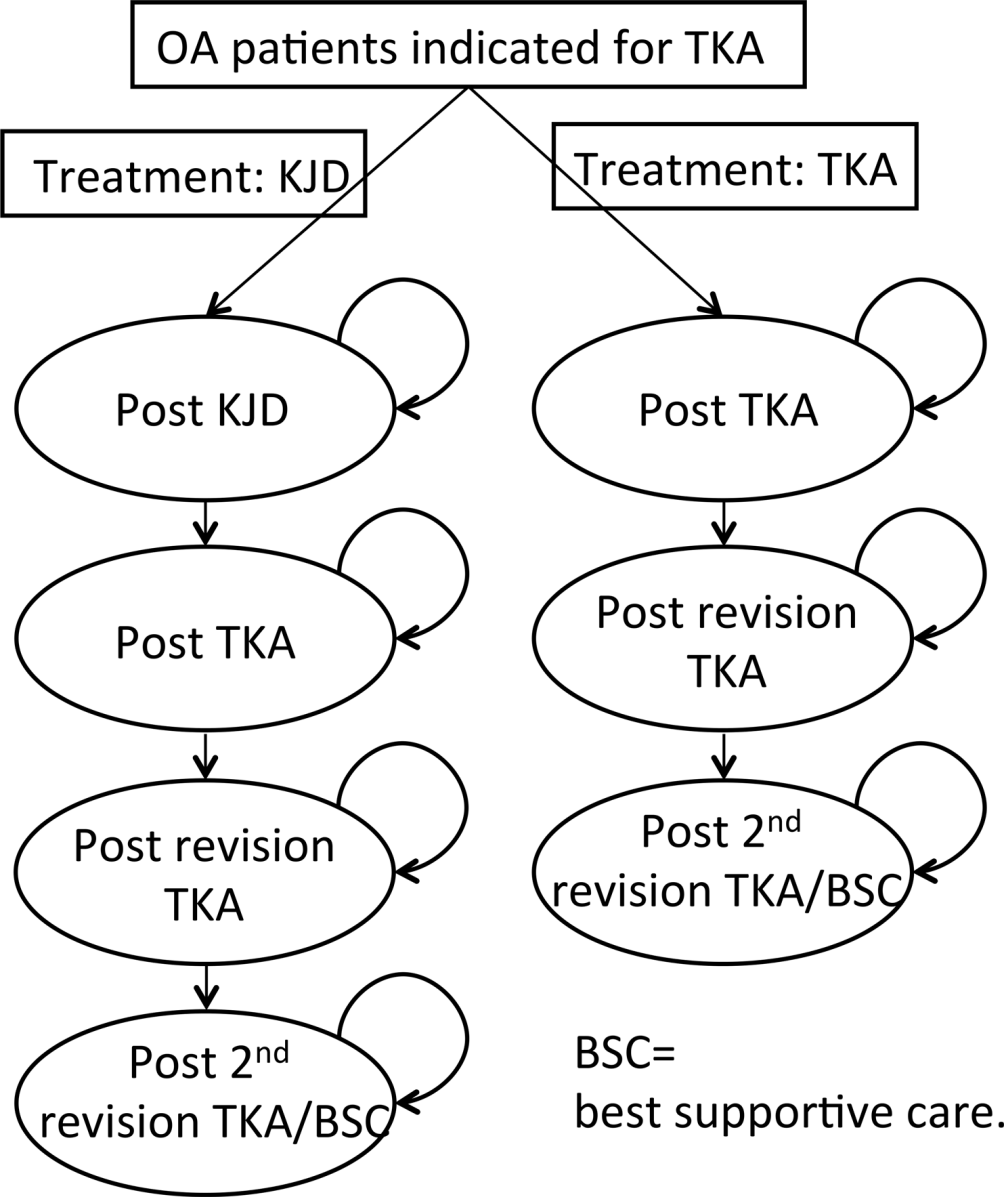
Overview of the health state model. Over the 20-year horizon analysis of the model patients are at risk of dying, so they can move to the absorbing state death from each other state. State death not shown in Fig BSC = Best Supportive Care.

To simulate a treatment strategy starting with TKA and a treatment strategy starting with KJD for patients of different age and gender and to extrapolate outcomes to long-term health and economic outcomes a simulation model was used. This individual patient Markov (or Health state) model evaluates the effectiveness of introducing KJD as first treatment compared to TKA over twenty years. This time horizon was considered adequate to capture the long-term impact (i.e. on revision surgery) of a treatment strategy starting with KJD as compared to TKA without going too much beyond the available follow-up data for the different surgical procedures, and a conservative approach as the longer time span the more chance on revision surgery. In the model a cohort of 200 patients was simulated to start with KJD (KJD strategy) and another (similar) cohort was simulated to start with TKA. After failure of KJD a TKA was performed (only for KJD arm) and after failure of TKA, revision TKA was performed in the model. When the revision TKA fails a second revision TKA or best supportive care was performed (Fig 2). Best supportive care (BSC) refers to care given after failure of revision surgery (if a 2^nd^ revision is not performed, i.e. analgesic therapy, a knee brace, or even knee arthrodesis). After the 2^nd^ TKA, it was assumed in the model that the patient remains in the ‘post 2^nd^ revision TKA/BSC’ state until twenty-year follow-up (or death, see below), since no data was available for time to failure after 2nd revision. Movement to the (absorbing) state of death is also possible in the model. Overall survival (life years left) was based on data stratified by gender and age for the Dutch population from the central bureau of statistics of the Netherlands, assuming the life expectancy of osteoarthritis patients to be equal to the general population (i.e. osteoarthritis and treatment specific mortality is assumed to be zero) [16]. In both cohorts patients are distributed over the health states according to the probabilities of failing of the different surgical procedures and the probability of dying over time with a cycle time (time interval over which a transition to another state can occur) of one year. The different health states (see Fig 2) are assigned a cost-value and utility value to obtain the total costs and quality adjusted life years over the total time horizon of twenty years.

### Cost calculation

Costs of KJD are calculated based on the actual/observed surgical equipment used, time spend on the surgery by the orthopedic surgeon and other personnel, hospital stay (on average 5 days) administrative hospital costs (i.e. overhead) and physical therapy costs (three months) of individual patients undergoing KJD, based on the presently available (limited and between hospitals variable) data. Additional non-surgical costs in the year of treatment are considered not different between both treatments and negligible compared to the surgery related costs. Resource use was valued using the Dutch manual for costing and Costs were measured in 2013 Euros [17]. Costs of TKA are based on tariffs for knee prosthesis cited by specific hospitals (Orthopedium Delft and Maartenskliniek, noting that cost may differ between different Dutch hospitals). Costs of revision TKA are based on expert opinion (SP, RvH, PvR, RC) and report from the Dutch health care insurer Achmea [18]. Costs as used in the analysis are shown in Table 2.

**Table 2:** Input data mean cost and utility per health state, point estimate with range as used in PSA.

**Costs**			
	**1^st^ year (Range)**	**Yearly thereafter (Range)**	**Source**
**Distraction**	€8.000 (€4.573-€12.370)	€0 (€0-€0)	Based on actual/observed costs with 10% markup
**TKA**	€12.000 (€8.405-€16.226)	€0 (€0-€0)	Based on tariffs cited by specific hospitals
**Revision**	€20.000 (€16.273-€24.106)	€1.000 (€376-€1.923)	Reference 18
**BSC**	€25.000 (€21.234-€29.069)	€100 (€38-€192)	Reference 18
**Utility**			
	**1^st^ year (Range)**	**Yearly thereafter (Range)**	**Source**
**Distraction**	0.73 (0.70–0.75)	0.82 (0.79–0.85)	EQ-5D RCT comparing knee distraction with TKA [10]
**TKA**	0.76 (0.73–0.79)	0.79 (0.76–0.82)	Reference 19–21
**Revision**	0.73 (0.70–0.76)	0.75 (0.72–0.78)	Reference 19–21
**BSC**	0.70 (0.67–0.73)	0.72 (0.69–0.75)	Reference 19–21

Since costs for TKA and revision TKAs are based on tariffs and no tariff yet exists for KJD, the costs based on actual observed costs were increased with a 10% markup. For the costs in the years after the procedure only for revision operation costs were assumed given the worse outcomes of these procedures and related care, for 2^nd^ revision these were assumed lower given the fewer treatment options available (Table 2), although this is conservative and may be an underestimation for costs of such a 2^nd^ revision. As such costs represent costs from a health care perspective (and not a societal perspective). Future costs were discounted using a constant annual rate of 4 percent [17].

### Utility estimation

Quality adjusted life years (QALY) takes into account both quantity and quality of life. For the different procedures QALYs were based on assigning a utility value for the year of the procedure (after) and one for the years thereafter the procedure. For KJD this utility value was based on the presently available data on the EuroQoL 5 dimension scale (EQ-5D) in a currently ongoing RCT, comparing KJD with TKA. [10] Utility for the other surgical procedures (in the year of the procedure and the years thereafter) are based on (changes in) scores after these procedures from the literature, as this contains more robust data for TKA than can be obtained from the before mentioned RCT [19–21]. For the utilities a discount rate of 1.5 percent was used [17]. Utility values as used in the model analysis are shown in Table 2.

### Analysis

The total number of operations, QALYs and treatment costs, and ICER’s expressing the costs per TKA saved, costs per revision operation saved, costs per 2nd revision/BSC saved, and costs per QALY as accumulated in the model per treatment strategy were calculated and the differences therein. This was done separately for gender and age categories (45–49, 50–54, 55–59, 60–64, 65–69). We used age and gender categories, since we also meant to get information on the 'best place/indication' for the treatment. Costs-effectiveness was also expressed using the cost-effectiveness plane. To obtain an (point) estimate as well as the uncertainty therein for the outcomes of the model a probabilistic sensitivity analysis (PSA) was performed. In this analysis for each individual patient in the simulation cohort a time to failure for KJD, TKA, revision TKA, and death is sampled and model outcomes are calculated. This simulation is repeated 5000 times for each age and gender category, in which also the cost inputs and the utility inputs are varied over a suitable range resulting in average estimates with 95% percent confidence ranges for the outcomes. The range for the average health state costs and health state utility in the PSA were varied using a uniform distribution for the cost inputs given the uncertainty in these input (Table 2). This analysis was performed for each gender and age category separately. Results are presented in cost-effectiveness acceptability curves. Apart from the PSA a deterministic sensitivity analyses (DSA), in which specific input variables are varied individually, was performed on:

- Time to failure for KJD (base failure time on data of KJD only, excluding ankle data)
- The cost of KJD procedure (using €10.000 instead of €8.000)
- Utility of KJD (using utility values in year of procedure and thereafter equal to TKA)
- The cost in years after revision and 2^nd^ revision surgery (set to €0 instead of €1.000 and €100 respectively)

## Results

**Fig 3:**
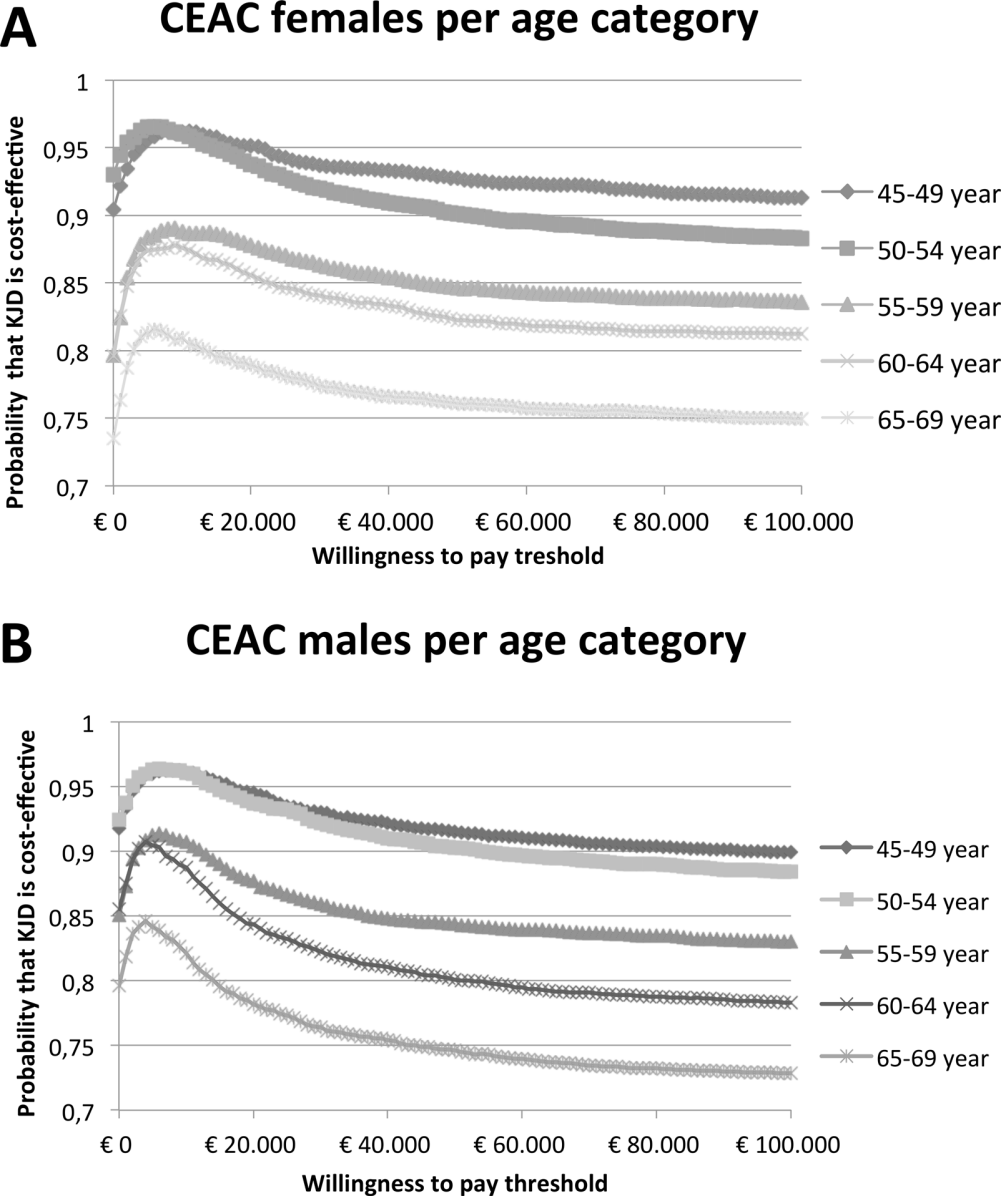
Cost-effectiveness acceptability curves. Females (A) and males (B) per age category.

**Fig 4:**
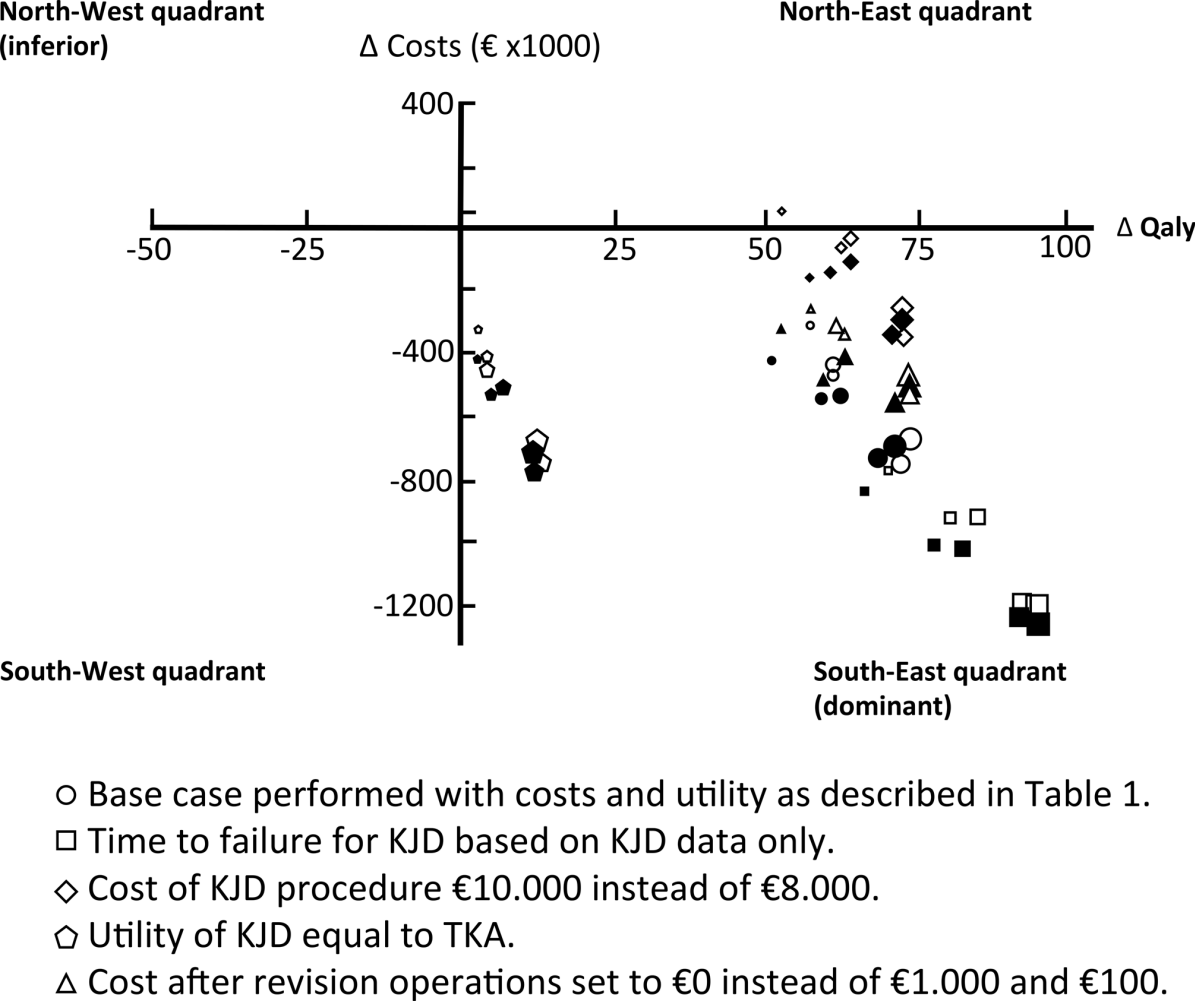
Deterministic sensitivity analyses. Females (open symbols) and males (filled symbols) per age category. The larger the symbol the younger the patient category.

Over 100 TKA’s are prevented by KJD over twenty years irrespective of age/gender category. Around 30 revision TKAs are prevented by KJD in the younger age groups (<55 years). Only few 2^nd^ revision operations are saved due to the low number of 2^nd^ revisions occurring over this (conservative but still reliable) time horizon. In general, starting with KJD saved costs. For detailed numbers of all age/gender categories see Table 3. Similar results in favor of KJD were seen for the average costs and QALY’s per person (Table 4). Furthermore, KJD strategy was dominant in about 80% of simulations (with only inferiority in around 1%) in the younger age categories (45–49 and 50–54) when compared to directly starting with TKA (see Table 4). At a willingness to pay a threshold of 20.000 Euro per QALY gained, the probability that KJD is cost-effective was already found to be over 75% for all age categories and over 90–95% for the younger age categories (Fig 3). The results of the DSA (Fig 4) also confirm the cost-effectiveness of this approach, with the KJD strategy on average being dominant (i.e. costs savings with QALY gain) in all scenarios in all age/gender categories except for females 65–69 years old for scenario two where the costs for the KJD procedure were increased from €8.000 to €10.000. The most cost-effective scenario was the one where time to failure was based on KJD data only (leaving ankle distraction data out).

**Table 3:** Differences between strategy starting with KJD and TKA after 20-years. **Overall result:** weighted average with weights according to the proportion of patients in the different gender and age categories undergoing TKA in the Netherlands [22].

Differences between strategy starting with KJD and TKA					
	No. TKAs prevented by KJD (95% CI)	No. of 1^st^ revisions prevented by KJD (95% CI)	No. of 2^nd^ revisions prevented by KJD (95% CI)	No. of Years on BSC prevented by KJD (95% CI)	Costs saved by starting with KJD (95% CI)
**Females**					
**45–49**	107 (93–121)	30 (18–43)	6 (1–13)	48 (1–103)	€681.740 (€-371.853–€1.649.483)
**50–54**	108 (94–122)	32 (20–44)	7 (1–13)	48 (2–100)	€744.004 (€-285.500–€1.715.557)
**55–59**	110 (96–124)	18 (8–28)	4 (0–8)	26 (-6–67)	€402.671 (€-618.273–€1.347.240)
**60–64**	113 (99–127)	18 (8–28)	4 (0–8)	24 (-5–62)	€421.703 (€-600.873–€1.370.455)
**65–69**	118 (104–132)	11 (3–20)	2 (-1-6)	14 (-8–44)	€297.486 (€-722.089–€1.231.619)
**Males**					
**45–49**	107 (93–121)	31 (19–44)	7 (1–13)	49 (1–104)	€729.266 (€-312.521–€1.768.484)
**50–54**	109 (96–123)	32 (20–44)	7 (1–13)	48 (4–100)	€753.401 (€-307.230–€1.709.497)
**55–59**	112 (98–126)	22 (11–33)	4 (0–9)	31 (-6-76)	€520.600 (€-521.532–€1.469.602)
**60–64**	117 (103–131)	21 (11–32)	4 (0–9)	28 (-4-68)	€542.960 (€-486.280–€1.498.937)
**65–69**	125 (110–139)	14 (5–27)	2 (-1-7)	15 (-7-48)	€413.259 (€-630.412–€1.381.296)
**Overall**					
	115 (101–129)	20 (12–31)	4 (0–9)	27 (-5-67)	€480.330 (€-550.213–€1.434.335)

**Table 4:** Average costs per person, average QALY per person with 95% confidence limits, and the proportion of the results of the simulations per quadrant of the cost-effectiveness plane after 20-years Overall result: weighted average with weights according to the proportion of patients in the different gender and age categories undergoing TKA in the Netherlands [22]. **Proportions CE-plane:** The North-East (NE) quadrant indicates that the KJD strategy is more effective but also more costly than the TKA strategy. A result in the South East (SE) quadrant means that KJD is dominant. A result in the South West (SW) quadrant means that KJD is less costly but also less effective, and a result in the North West (NW) quadrant means that KJD is less effective and more costly (inferior).

	Strategy starting with TKA		Strategy starting with KJD		Proportions CE-plane			
Category	Average Costs per person (95% CI)	Average QALYs per person (95% CI)	Average Costs per person (95% CI)	Average QALYs per person (95% CI))	%NE	%SE (dom)	%NW (inf)	%SW
**Females**								
**45–49**	€16.700 (€12.600-€21.100)	13.2 (12.7–13.7)	€13.200 (€9.300-€18.000)	13.6 (13.1–14.1)	8.6%	80.7%	1.0%	9.7%
**50–54**	€16.600 (€12.700-€21.200)	13.0 (12.4–13.6)	€12.900 (€9.100-€17.600)	13.4 (12.9–13.8)	7.0%	79.9%	0.9%	12.2%
**55–59**	€14.700 (€10.900- €19.200)	12.7 (12.1–13.3)	€12.700 (€8.900-€17.400)	13.0 (12.5–13.6)	16.3%	66.4%	3.7%	13.7%
**60–64**	€14.600 (€10.800-€19.100)	12.2 (11.5–12.9)	€12.500 (€8.700-€17.200)	12.5 (11.8–13.1)	16.1%	63.6%	3.6%	16.7%
**65–69**	€13.600 (€9.800-€17.900)	11.3 (10.5–12.2)	€12.200 (€8.400-€16.500)	11.6 (10.8–12.4)	19.9%	53.5%	7.2%	19.4%
**Males**								
**45–49**	€16.900 (€13.000-€21.500)	13.1 (12.5–13.6)	€13.300 (€9.300-€17.900)	13.5 (13.0–13.9)	6.7%	80.7%	1.1%	11.5%
**50–54**	€16.700 (€12.800-€21.100)	12.7 (12.1–13.3)	€13.000 (€9.300-€17.700)	13.1 (12.6–13.6)	6.9%	78.7%	1.2%	13.3%
**55–59**	€15.300 (€11.400-€19.700)	12.3 (11.6–12.9)	€12.700 (€8.900-€17.300)	12.6 (11.9–13.2)	12.5%	68.4%	2.7%	16.4%
**60–64**	€15.100 (€11.400-€19.600)	11.5 (10.6–12.3)	€12.400 (€8.600-€16.800)	12.2 (10.9–12.5)	15.2%	64.6%	4.0%	16.2%
**65–69**	€14.100 (€10.200-€18.400)	10.2 (9.3–11.2)	€12.000 (€8.200-€16.700)	10.5 (9.5–11.5)	15.6%	55.5%	5.8%	23.1%
**Overall**								
	€14.100 (€11.100-€19.300)	12.0 (11.2–12,7)	€12.500 (€8.700-€17.100)	12.3 (11.5–12.9)	14.6%	64,8%	4.0%	16.6%

## Discussion

This study found that when patients with generalized knee osteoarthritis are first treated with KJD before TKA, this leads to delay of revision TKA surgeries, and effectiveness in terms of quality adjusted life years. Moreover starting with KJD saves costs. This resulted in a very high likelihood for the KJD strategy to be cost-effective, in specifically the younger age categories (45–54 years). Even if the costs for KJD were increased to €10.000, KJD still dominates TKA (except in females aged between 65–69).

Less favorable outcomes on cost and effects were seen for the older age categories. This makes sense because it is less likely that elderly need (more costly) revision surgery during their lifetime, consequently these operations cannot be prevented by KJD when performed at a later age (i.e. 65–70 years). Nevertheless, in all age categories the KJD treatment strategy was found to be dominant or cost-effective. Given the sizable burden of osteoarthritis especially in the ageing and obese population, KJD can substantially contribute to the improvement in quality of life in this population. Females benefit slightly less than males as a result from the slightly more benefit (in terms of time to failure) they have from TKA.

Early assessment of medical technology is an important step. As shown by Steuten et al.[23], an early HTA analysis can provide critical insights for technologies in development using this decision analytic approach. An important part of such an early technology assessments is often a cost-effectiveness (CE) analysis. Such CE analyses give insight into whether new technologies (such as in this case KJD) have potential in terms of cost and clinical effects (i.e. value for money) and also in which situation(s) (i.e. setting and patient population). In that way it can be determined what the patient and social impact likely will be and also what the likely returns on investment in further development of the new technology would be [23].

Our analysis made a number of assumptions and clearly has some limitations. First assumptions were made regarding the costs of the KJD procedure, due to lack of an available tariff (as used for the TKA procedure). However, for the costs of KJD, data on medical consumption (i.e. personnel material etc.) was used from the clinical setting where the frame was used and a markup of 10% was used to obtain comparable cost data, which is an often-used way of determining tariffs. Other costs were based on tariffs or estimations of tariffs from clinical experts and a health insurer. As such the perspective of the cost-effectiveness analysis was that of the health care system. This analysis did not consider high tibial osteotomy or unicompartimental knee prosthesis as part of the treatment standard, since these procedures are performed solely in patients with unicompartimental disease, whereas the KJD patient population concerned patients considered for an initial TKA with (mainly) bicompartmental knee osteoarthritis.

As we estimated costs from the healthcare perspective, no productivity costs were included in the analysis. This can be seen as a limitation, since patients with knee osteoarthritis requiring surgery often have cost due to lost productivity [24]. However, hospital treatment costs comprehend most of the cost and adding costs due to lost productivity would probably have led to similar results as we do not expect that during the procedures and in the years after effective procedures these costs are significant different between TKA and KJD. This is expected even if the loss of labor during the six weeks distraction for some patients will be included, since also TKA patients will not regain labor directly after surgery. Although patients receiving KJD overall have more operations (extra KJD procedure for removing of the distraction frame) the higher number of revision surgery and/or complications at earlier age will lead to higher loss if productivity. Another limitation was that the survivorship of knee implants was based on historical data. This could have led to an underestimation of the current survivorship given the technological advancement in recent years. However, this may also be the case for KJD as a relative novel technique, still further improving in its technology (e.g. pin tract infections are significantly reduced, treatment is changed from three months in the first study toward six weeks in the present studies) and with that potential better clinical outcome. In addition, since we used a twenty-year time horizon, this could lead to an underestimation of lifelong revision TKA surgeries and costs, since it is reasonable to assume that after this twenty-years the number of TKA failures increases and therefore more revisions might be prevented by first performing KJD. Furthermore one has to bear in mind that KJD is not the final stadium for a patient. If, after KJD, patients still have knee pain (or even an relative increase in knee pain) another good treatment option, namely TKA, is available and this step is then easily made. After TKA, only less optimal treatment options are available which makes the step from TKA to revision TKA not as easy [25]. After TKA about 80% of the patients is satisfied, meaning that as many as 20% remains to have persisting problems [26,27]. This reflects itself in the fact that the number of patients dissatisfied with the outcome after TKA is higher than the number of patients requiring revision.

Our model also had some other limitations. Since we meant to obtain information on the 'best place/indication' for KJD, we decided to report results per age and gender category. However, for KJD and revision TKA no (sufficient) data was available for specific estimates of time to failure per gender and age category. Therefore the same input for time to failure was used for all gender and age categories for KJD and revision TKA in the model (Fig 1). However, for life expectancy and time to failure for TKA specific estimates per gender and age category was available and used in the model

This is the first analysis systematically evaluating the likely health gains and costs (savings) of implementing KJD as new treatment for osteoarthritis in current clinical practice using the best available data present. Although results are inherently uncertain this analysis shows high potential in effectively postponing TKA and preventing revision surgery. This will definitely improve quality of life of patients with a very high probability of cost-effectiveness. Additionally, in case of KJD the patient’s own knee is saved, whereas a TKA is at the expense of the original joint.

In conclusion, the findings suggest that a treatment strategy starting with knee joint distraction for osteoarthritis shows large potential for being a cost-effective intervention, especially in relatively young patients. Future studies should focus on this population first.
